# Cultural responses to the COVID-19 crisis in Greece: The first wave (March–May 2020)

**DOI:** 10.1177/00472441221141957

**Published:** 2023-01-16

**Authors:** Panagiotis Zestanakis

**Affiliations:** University of Hamburg, Germany

**Keywords:** COVID-19, emotions, Greece, lifestyles, media, modernity, politics

## Abstract

This article examines cultural responses to the COVID-19 pandemic in Greece during the first wave in spring 2020 approaching such responses not only as resulting from the fear of the virus but also as outcomes of larger historical processes. Analysing media material, surveys and discourses by politicians and health specialists the article argues that although Greece had a limited number of cases, most Greeks embraced the lockdown seeing COVID-19 as a threat. The government’s communication strategy highlighted popular values such as family and downplayed the class dimensions of the pandemic. This strategy proved effective and increased the popularity of the government and health experts at that stage.

Frankema and Tworek (2020: 335) argue that pandemics are central to global history. They question the foundations of society, the sustainability of its material basis, expertise, social codes and behavioural norms. The COVID-19 pandemic showed us how the path of a virus can interact with globalisation. This article focuses on Greece and explores, from a historical perspective, cultural responses during the first lockdown (March–May 2020). It discusses the government’s communication management and stresses how the acceptance of measures contributed to the popularity of politicians and health specialists. The New Democracy government under Prime Minister Kyriakos Mitsotakis presented itself as a safeguard of Greece’s modern identity and employed traditional cultural references, such as the family representing a locus of safety, to cultivate emotions of national pride. A second wave began in September 2020, leading to a new lockdown on 7 November 2020 which lasted until 15 May 2021. This article does not juxtapose the two lockdowns but includes comparative remarks that help to better understand the initial stage of the COVID-19 pandemic. The analysis is culture-focused and not economy-focused. The economy was a crucial dimension of the COVID-19 pandemic; however, in the first wave, public discourses centred on health. The shock, the relatively limited duration of the first lockdown and the conviction that the pandemic would not last long – approximately 40 percent of the Greek population believed that normality would return within 2 months (Georgakopoulos, 2021: 37) – rendered the economy a side issue.

Culture is a set of values, assumptions, beliefs, orientations to life, behaviours and procedures shared by communities of people (Matsumoto and Juang, 2003: 5–9; Spencer-Oatey, 2008: 3). Cultural responses are embedded in a specific (in this case national) cultural framework and express enduring transformations. The experience of the first wave in Greece was inscribed in larger historical processes. The cultural responses exceeded the ‘rally-round-the-flag’ effect (the phenomenon whereby in crises, governments benefit from a – usually ephemeral – rise in public support; Mueller, 1970), which affected most societies in the first wave (Cunningham, 2020). For the recently elected New Democracy government it provided a space to discuss the construction of a ‘success story’ which largely concerned the representations of politicians and epidemiologists as heroes. This presented an opportunity to discuss perceptions of the family, masculinity and intergenerational relations and their interconnections with politics.

By May 2020, even cadres usually critical of the Government such as the ex-undersecretary of health Pavlos Polakis acknowledged that Greece had reacted well (Interview with Alpha TV, 2020). The opposition presented itself as a contributor to the success story, claiming its own share of the national pride, which in turn invigorated the Government’s communication agenda. At that time, the Government enjoyed popularity far beyond its conservative power base. In April 2020, 73 percent of voters took a positive view of Mitsotakis (Metron Analysis, 2020).

The COVID-19 pandemic was a novel crisis. Even historians had forgotten the closest similar experience: the Spanish Flu outbreak of 1918 (Honingsbaum, 2018). As Schwab and Malleret (2021) argue, the COVID-19 pandemic shocked the world, showing that even rich countries were unprepared for such a threat. The pandemic fed existential anxieties; demonstrated the limits of global governance; highlighted how people from different classes are exposed to danger in dissimilar ways (e.g. working-class people were often in jobs that meant they could not work from a distance); altered habits regarding communication through the increase of online services; and strengthened big enterprises, which appeared better prepared to face such a challenge. For Demertzis (2020), the COVID-19 pandemic generated anxiety and fear. People faced interruption to their everyday routines, likely to be the harshest since the Second World War. The use of mass burials in the United States, for example, was reminiscent of hardships unknown since the 1940s. People did not know how to deal with such an event. Although the pandemic was a highly mediatised phenomenon, reliable information was limited. Public spaces were evacuated. As people were forced to stay at home, consumption of information from social networks increased by 55 percent in Spain, 30 percent in Italy, 18 percent in the United Kingdom, 14 percent in France and 11 percent in Germany (Moreno et al., 2020: 3). The pandemic traumatised certain sections of the population. The elderly died in hospitals or alone at home, unable to see their children or friends. Death became a private issue. The pandemic, which initially touched well-connected areas of the planet (Snowden in interview to Willik, 2020), demonstrated the fragile character of globalisation and led to the fast de-touristification of societies as governments introduced strict border controls. This impacted negatively on the Greek economy: Tourism represented 20.8 percent of the Greek GDP in the 2019 and employees in tourism represent 21.7 percent of the total workforce (SETE, 2019).

The public’s placid acceptance of anti-pandemic measures can be attributed to the manner in which authorities handled emotions of fear and anxiety, turning them into sympathy with their policies. Emotions are historically and geographically located, bundled with together with reason and sensation; they acquire different meanings through time (Boddice, 2017: 11; Hunt, 1989: 7; Wierzbicka, 1999: 32). Emotions are historicised and de-essentialised cultural processes stimulated by sources such as media texts (Ahmed, 2004; Scheer, 2012) and emanate from interactions between individual and social situations (Demertzis, 2013: 7). Societies set up emotional regimes within which certain expressions (e.g. respect for authority) are induced and maintained (Oatley, 2004: 15–18; Reddy, 2001). Emotions represent a privileged area for accounting for the relations between rules, individuals and the social order and cannot be explained by social norms as there are no general agreements on norms in societies (Barbalet, 2002: 2).

Pride is an emotion of pleasure springing from one’s own achievements, the achievements of the community to which one belongs (e.g. the nation) or from qualities seen as admired. Pride is considered a marginal emotion and attracts less scholarly attention than fear, anxiety and happiness (Lewis et al., 1989; Tracy and Robins, 2007). In Greece, especially since the mid-1990s, national pride has often been derived from the idea that the country was modern. In the economic crisis of the 2010s, Greece’s modern identity was contested. During the COVID-19 pandemic, emotions of national pride sprang from the fact that the Government offered an appealing narrative, stressing that the effective pandemic response signalled Greece’s return to a lost modern identity. During the first wave, the Greek Government’s communication strategy owed much of its impact to the way it attenuated negative emotions and evoked sentiments of solidarity and national pride, while downplaying the class dimensions of quarantine and other anti-pandemic measures. In mid-April 2020, the historian Yuval Noah Harrari argued that Greece had done excellent work in limiting the pandemic. Greece had only approximately 2,300 COVID-19 cases in late May 2020. Pro-government media highlighted such comments from international commentators (e.g. Ekathimerini, 2020), triggering emotions of pride. This was important, as during the economic crisis of the 2010s, the international media had criticised Greeks as spoiled and counterproductive (Mylonas, 2020: 87–152). Greeks traditionally believe that things will turn out badly: only 13, 21 and 28 percent believed that things were going well in surveys in April 2017, April 2018 and April 2019, respectively. In contrast, in April 2020, 86 percent believed that the country was heading in the right direction (Georgakopoulos, 2021: 35).

There is an extensive body of literature on the Greek crisis of the 2010s (e.g. Siani and Davies, 2017; Tziovas, 2017). Briefly, income reduced, unemployment skyrocketed and collective happiness fell. Expressing anger and disappointment, SYRIZA (the Coalition of the radical left) promised to end austerity and saw its popularity surge, winning two elections in 2015 which resulted in governments with the right-wing party of Independent Greeks (ANEL) which continued austerity. SYRIZA remained in office until July 2019 when New Democracy came to power. SYRIZA showed itself ineffectual when it came to managing crises, culminating in the disaster of 23 July 2018, when authorities failed to control wildfires in eastern Attica and 102 people died. At that time, the New Democracy party, and media friendly to it, emphasised this inefficacy, presenting New Democracy as a safeguard of the country’s modern identity. The championing of modernity also marked the communication management of the first wave.

The crisis of the 2010s challenged Greece’s modern identity. Greece has often been considered a society sitting at the intersection of tradition and modernity. The perception of inadequacy cultivated an idiosyncratic nationalism that drew on the country’s glorious past (Mouzelis, 1978). A currently popular approach describes periods of modernisation followed by counter-modern historical phases (Kalyvas, 2015). A similar argument sees Greece as a country that remains modern in its own controversial manner (Voulgaris, 2019). The climate changed in the 1990s and the 2000s, which were marked by pro-modernisation and pro-globalisation optimism. The dominant narrative focused on achievements, such as rising living standards and adoption of the Euro. Although traditional agents such as the Orthodox Church remained powerful (Verney, 2002), the notion that the country was underdeveloped faded (Voulgaris, 2019: 132–3). In early 1990s Spain, modernisation was associated with international events such as the 1992 Olympic Games in Barcelona and the Seville Expo ‘92 (Palacio, 2020); in Greece, modernisation revolved around the 2004 Olympic Games in Athens, infrastructure projects such as the Rio-Antirrio Bridge and participation in the Eurozone. This trend was disrupted by the 2010 crisis, an event that historically and psychologically ended *Metapolitefsi*, the period which started after the 1974 political changeover (Liakos, 2019: 20). Greece experienced virtually no respite between the 2010 economic crisis and the COVID-19 health crisis. The New Democracy government presented its in-time reaction to the pandemic as part of a return to modernity, reinforcing critique of its predecessor which it replaced after the July 2019 elections when New Democracy won 39.85 percent of the vote compared to the 31.53 percent won by SYRIZA.

In Greece, the early implementation of anti-COVID measures and the fact that the pandemic began in winter, when the country receives limited tourists, contributed to the low number of cases given that the authorities closed the borders on time. The public embraced the measures and, in contrast to other countries, such as Germany or the United States, there were no demonstrations against them. Greeks did not comply with the measures out of fear of penalties. The fine for unauthorised movement was 150 Euros (300 Euros in the second lockdown), which is less than most fines for breaking the Highway Code in a country where violations of the latter are common. The Greek aversion to risk, which is one of the highest worldwide (Anagnostakos, 2021: 48), is a more convincing explanation than fear of penalties or the police. The adoption of measures also expressed Greek citizens’ desire to be re-included in narratives of modernity and challenged the image of the counter-productive and spoiled nation that was cultivated in the international media – and that Greeks often endorsed – in the 2010s (Kalantzis, 2015; Mylonas, 2020: 160–76). In surveys held in September 2020, large majorities stated that hygiene measures should be respected: 95.5 percent stated that they washed their hands very often, 85.1 percent wore masks when required and 79.7 percent respected social distancing measures (Georgakopoulos, 2021: 41).

The COVID-19 pandemic which began in China in December 2019 and ravaged Italy in February 2020,^1^ reached Greece on 26 February when the first case was recorded. The first patients were people who travelled to Italy; given the proximity between the two countries, the Government took measures quickly. This determination made a difference compared to countries such as the United Kingdom, where the government left about seven weeks between the first infection, recorded on 1 February, and the decision for a general lockdown, which was taken on 23 March (Newton, 2020: 505). In Greece, by 10 March 2020, there were 89 confirmed cases. Until then, the measures had concerned the cancellation of cultural events and the closure of some schools. On that day, the government closed all educational institutions. On 13 March, it ordered the closure of cafes, restaurants, bars, museums, malls and sports venues, and on 16 March it closed all shops except those catering to essential needs. On 23 March, the Government imposed restrictions on movement. People were allowed to leave their homes only to go to their workplace or to essential shops, to visit people requiring help and for limited physical exercise, all only after requesting permission via text. Between 23 March and 20 April 2020, the police reported only 46,420 ‘unnecessary outings’.

Respect for family bonds certainly influenced popular emotional responses. The virus threatened the elderly, who are well respected within the Greek family. The images coming from countries heavily struck by the first wave such as Italy which showed elderly people dying *en masse* frightened a society in which the trust of state services is moderate. Young people – who would otherwise be more likely to ignore the measures as the virus primarily threatened older and chronic patients – often have deep emotional bonds with the elderly members of their families, strengthened through regular family meetings. Because of the close-knit structure of the Greek family and the crisis of the 2010s, many middle-aged people still live with their parents, who belong to age groups considered at high risk. According to Eurostat (2020), in 2019, Greeks typically did not leave the family until they were past the age of 30. The weak welfare system means that it is the grandparents who often take care of the children when their parents have to work, and this further strengthens the bonds between the elderly and their grandchildren. It is no coincidence, therefore, that when vaccinations for the elderly began in early 2021, public gatherings of young people became more common (and certainly more visible). Greece is a moderately individualistic society by European standards (Rothwell, 2016: 59); the value of the health of the community is recognised as important.^2^ Furthermore, Greeks traditionally avoid risky situations such as stays in hospitals (Anagnostakos, 2021: 48). As welfare structures are mediocre, it is the family that often provides socioeconomic security to its members. Greece spends approximately 10 percent of its total general expenditure on social protection, about half of the EU-27 average (Eurostat, 2018). According to the OECD (2017), only 31 percent of Greeks trust the state’s health services, with affluent families opting for private hospitals. In addition, since the 1980s, middle-class families have increasingly offered their young members pleasant consumer experiences that the latter could not otherwise have afforded. Consumption strengthened intra-generational solidarity. This condition perpetuated relations of care which were expected to be mutual (Panagiotopoulos, 2018: 177–83). Children support their parents when they need help. This culture of mutual care underpinned intergenerational solidarity in the initial stages of the COVID-19 pandemic.

These close relations have not always prevented the exposure of the older generations to risks. The July 1987 heatwave with more than 1000 victims, mostly older people living alone in Athens, is a noticeable example, but this traumatic event was quickly redacted from public memory (Markatas, 2010). In reaction, families started to install air-conditioning units to protect their older members (Zestanakis, 2020: 54). Hence, although from the 1980s onwards Greek society became more individualistic (Tziovas, 2003: 46) and increasingly trusted state institutions, there was no unanimous transfer of trust from family to the state. Unsurprisingly, during the COVID-19 crisis the protection of family emerged as a priority, motivating people to endorse the measures.

Surveys held in mid-April 2020 showed Greeks maintained hygiene rules and spent time at home with family, communicating by telephone or via the Internet, cooking and watching television and/or Netflix. As in other countries, people started using online stores and reduced their physical exercise (*Ta Nea*, 2020a). About 80 percent of young people saw the quarantine as a period for introspection, which induced uncertainty, fear, anxiety and pessimism (Chtouris and Zissi, 2020: 53–8). In such a climate it was impossible to organise political protest. With the exception of the far-right Golden Dawn, now condemned as a criminal organisation, which articulated an unpopular anti-mask rhetoric, the government’s choices enjoyed consensus. SYRIZA, the main opposition party (with support estimated at 22–25 percent in surveys held in early 2020), criticised certain measures such as the cancellation of the Patras Carnival as unnecessary but did not organise protests. The argument proposed by philosopher Giorgio Agamben (2020) that the Italian government saw the COVID-19 pandemic as an opportunity to establish a permanent state of emergency did not reach the Greek Left.

SYRIZA’s consent to the measures was based on the promise that the government would be made to apologise for deficiencies in the health system later on. The slogan «μετά θα λογαριαστούμε» (we will resolve our differences after the crisis) became a hashtag in SYRIZA-friendly social media but its impact was weak, likely because the SYRIZA-ANEL government had continued austerity politics and not impressively improved public health. According to Euclid Tsakalotos (2020), the Minister of Finance between July 2015 and July 2019, Greece had 430 acute-care beds in 2015 and 560 in 2019. Research by Avgeropoulos (2021), based on interviews with health workers, shows that after a decade of austerity, the COVID-19 pandemic found the health system ill-prepared. Avgeropoulos also shows that state services neglected vulnerable groups such as homeless people, drug-addicts or refugees living in camps. Immigrants of colour experienced police controls frequently (Karathanasis and Kallianos, 2022). The opposition failed to articulate a coherent discourse that gave prominence to these problems. For political scientist Ivan Krastev (2020: 24–5), everywhere in Europe opposition parties lost their role in the pandemic. Europeans trusted their governments more than Asians and Americans, but there are examples to the contrary; Spaniards and Italians felt disappointed by their governments (Alonso, 2020; Georgakopoulos, 2021: 36).

The communication management of the COVID-19 crisis thus concerned modernity and how in-time reactions to threats confirmed Greece to be a modern state. Greece has faced limited situations of genuine danger since the return to democracy in 1974. While inequalities remained noticeable even in the prosperous 2000s (Balourdos and Yfantopoulos, 2007), extreme poverty was limited and everyday life no more hazardous than in most west European countries (Liakos, 2019: 409–62). A war with Turkey seemed probable in 1996 (Athanassopoulou, 1997), but beyond that, despite moderate political crises (e.g. that of 1989–90 when three elections were held in less than one year), post-1974, Greece experienced stability. In the years around the 2004 Olympic Games, discourses on a modern Greece flourished. The idea that the Games positively influenced Greece’s image, reinforcing its national self-confidence, continued for years (Georgiadis and Theodorikakos, 2015: 3–4). This sense of pride got hurt during the economic crisis of the 2010s.

The government argued that the successful response during the first wave of the COVID-19 pandemic confirmed the country’s return to modernity and created a success story around the concept of effectiveness: Greece was again a modern country, and its inhabitants should feel proud of this. This was in tune with New Democracy’s agenda as an opposition party between January 2015 and July 2019, when it highlighted its supposed ability to deal better with crises and represented itself as a force of modernity. This rhetoric reached its peak after the July 2018 wildfires in Attica when it argued then that the government was ill-prepared and put human lives in danger. The ineffective management of the disaster in terms of government communication assisted New Democracy’s agenda. The government insisted that there were no victims for hours after the disaster. When the reality became apparent, the government argued that authorities had satisfactorily reacted and that, given the extreme weather conditions, the disaster was unavoidable. This argument, which may well have satisfied voters in the first post-war years when Greece was a very poor country, and such disasters were seen as inevitable, was not accepted in 2018. As sociologist Panayis Panagiotopoulos (2013: 190) argues, in postwar Greece, the management of disasters emerged as a marker of modernisation. By the 1990s, it was believed that disasters should be anticipated and confronted and concepts such as ‘organisation’ and ‘prediction’ gained ground as cyphers of modernity. From the vantage-point of emotions, people could feel safe and proud of their state as the latter could prevent its citizens from being exposed to danger. In the 2018 wildfires, politicians of the SYRIZA-ANEL government avoided visiting the area of the disaster. When Prime Minister Alexis Tsipras finally made an appearance he arrived early in the morning to avoid encountering the inhabitants. The government blamed the residents of the area themselves who had constructed illegal buildings and blocked access to the sea. The president of ANEL and minister of national defence Panos Kammenos quarrelled with them on camera. This imperious demonstration of authority increased popular dissatisfaction. The reaction of the state was evaluated as unsatisfactory by 81.1 percent of the public; 77.6 percent thought the government was responsible for the disaster (Marc, 2018: 4–5). The impact of this in the 2019 elections was not negligible (Kerin, 2019). Memories of this inefficiency survived in 2020: In surveys conducted after the end of the first wave in summer 2020, 52.9 percent believed a SYRIZA government would have managed the COVID-19 pandemic worse.^3^ Due to its citizens’ belief that the state reacted quickly, the government’s popularity increased.^4^ This reaction strengthened feelings against SYRIZA (the ‘anti-SYRIZA syndrome’) as the government appeared well-prepared in the management of crises and effective in communicating this preparedness to the media (Moschonas, 2022). A critical communication choice was to inspire an understanding of the measures by citizens; given the values of Greek society, an appealing argument was that youngsters had to protect the elders. Mitsotakis propounded this on television. Another key individual was Sotiris Tsiodras, professor of medicine at the University of Athens. Tsiodras participated in the committee of experts, which proposed measures, and was a media spokesperson in addition to the Deputy Minister of Civil Protection, Nikos Chardalias. The Minister of Health, Vassilis Kikilias, appeared less in the media.

Mitsotakis, Tsiodras and Chardalias attempted to provide the impression that they could handle the crisis as technocrats while remaining approachable. They targeted the public’s emotions and aimed to transform feelings of insecurity and fear about the COVID-19 pandemic into sympathy for the government. In his address to the Greek people on 11 March 2020, Mitsotakis employed a communication model based on a frank discussion of the difficulties of policy-making, which was used by various leaders worldwide such as German Chancellor Angela Merkel and Finnish Prime Minister Sanna Marin (Newton, 2020: 509). He attempted to establish an intimate relationship with the public, using friendly tones to explain how young people should behave. This choice was compatible with his earlier profile, as he has in the past attempted to establish a youth-friendly identity, playing sports on camera, uploading photos of his family life on Instagram account or appearing in talk shows.^5^ The use of Instagram, a medium that young people prefer (Leaver et al., 2020: 19–23), shows that Mitsotakis was interested in addressing young audiences (a group usually not favourable to a country’s conservative party) using their own means of communication. He struck a paternal note, mentioning habits such as watching movies, a rare tactic for Greek prime ministers who prefer more formality: Modernity was communicated through a ‘cool’ vocabulary compatible with current social media aesthetics investing in traditionalist values such as family. This concurrent investment in modern and traditionalist referents enabled him to reach a wide audience.

This discourse romanticised the experience of quarantine and designated COVID-19 as a ‘great equaliser’ (Berkhout and Richardson, 2020: 3), sidelining the class dimensions of its effects. It was after easier for the affluent and those with bigger houses to talk up quarantine. The first broadcast of the Secretariat of Civil Protection, with instructions on how people should behave, was filmed in a roomy house and not in a flat.^6^ This disregard of socioeconomic inequality had no political cost in the first lockdown and rather limited cost in the second. This could be attributed to the rally-round-the-flag effect, the relatively short duration of the confinement, the support that the media provided to the government, and the inability of the opposition to offer an alternative narrative.

This vocabulary designated family and home as metonyms of safety, sidelining collateral effects such as the probability of increased gender violence (Boyce Kay, 2020) or the re-masculinisation of the public space because of men leaving the home about twice as often as women in Greece (Georgakopoulos, 2021: 37). International experience shows that the lockdowns led to an increase in anxiety and the consumption of alcohol (Polakovic, 2020), creating ideal conditions for rising family violence. Google searches related to domestic violence increased worldwide (Grammatikaki, 2021: 68). It is likely to be no coincidence that Greece witnessed a wave of femicides during and after the pandemic’s second wave. The Greek experience confirms that the COVID-19 pandemic was a bad time for many women (Berkhout and Richardson, 2020; Lewis, 2020).

Images focused on family, especially through references to inclusive masculinity that embraces the expression of emotions of tenderness and care, until recently primarily associated with femininity (Anderson, 2009), played an important role in the communication management of the pandemic. Mitsotakis stressed his identity as a family man, an impactful choice in a society that has endorsed the value of the nuclear family. Mitsotakis highlighted this identity via posts of himself with his children (e.g. 12 April 2020) and more eloquently through a photo showing his children sleeping together at home (24 March 2020). The caption – ‘They stay at home, I am at the office’ – stressed his identity as a protective father and implicitly identifies the family with the nation, highlighting Mitsotakis’ willingness to protect them both. Such posts aimed to cultivate expectations of security around Mitsotakis’ willingness to act as a responsible leader. This performance of responsible masculinity aimed to trigger emotions of pride, identifying the prime minister with ceaseless work. The post aimed to send the message that although most people doing office jobs can work from home, the prime minister ceaselessly worked to protect the nation, exposing his health to danger. This gave his sympathisers a sense of pride based on the contrast between a culture of work (represented by the government) and one of lack of effort expressed by its political opponents. Interestingly, this communication style boosted the popularity of Mitsotakis even among left-wing voters: in a June 2020 survey, 25.1 percent of SYRIZA’s voters preferred Mitsotakis to the leader of their party (Opinion Poll, 2020: 12). The success of this communication strategy is corroborated by the fact that SYRIZA’s leader adopted it. Tsipras, a father of two children, started uploading photos of family moments to Instagram (e.g. 5 April 2020; 13 September 2021) and embraced the kind of traditionalist imagery that he had rejected earlier in his career.

Such developments show that masculine identities drawing on the ideal of nuclear family are influential. Mitsotakis’ and Tsiodras’ communication styles shared common features, employing emotional vocabulary, highlighting the need for solidarity . Tsiodras shredded tears during a press interview. Such vulnerability is uncommon for a public figure in a country where crying is incompatible with hegemonic masculinity. The media portrayed Tsiodras as a figure who possesses a talent for explaining science to lay audiences. The media also positively viewed his explanation of the measures to Romani people in person when he stressed that minorities should not become the scapegoat. As discussed, this approach did not characterise the government’s policy regarding COVID-19 and ethnic or social minorities in general. Health crises intensify the social boundaries between groups as people attempt to distance themselves from those perceived to be unhealthy, stereotyping already stigmatised groups (Dionne and Turkumen, 2020: 214). Tsiodras demonstrated a willingness to minimise such reactions, and this activity boosted his popularity: 94 percent had a positive opinion of him in April 2020 (Ethnos, 2020). This imagery energised liberal audiences as the state appeared ready to protect the whole population. Tsiodras emerged as an ‘epidemiologist-cultural hero’ (Lynteris, 2015) inspiring emotions of solidarity and national pride. Seeing Tsiodras’ identity from the vantage-point of a conservative audience, this sympathy likely springs from the fact that, unlike many scientists, Tsiodras is religious, a father of seven children who occasionally chants in churches. This pious masculinity boosted his popularity in a society where 56 percent see religion as important (in most West European countries this figure is between 10 and 20 percent; Pew Research Center, 2018). Chardalias employed a sterner communication style, but in speeches and social media he underlined his attachment to family and religion, for example uploading a photo from his daughter’s birthday on Instagram on 20 May 2020 or appearing unhappy when announcing that religious ceremonies had to be cancelled. Such posts showed that family remained a crucial referent in a condition that favoured individual living and isolation such as the COVID-19 pandemic. They promoted the idea that traditional values could be part of a successful reaction to a threat, corresponding to a modern state of which society could be proud. Mitsotakis and Chardalias used Instagram’s ability to visualise and transmit such messages, energising positive emotions. Given the popularity of religion and family, such visual stimuli enabled the government to appear as a political power representing the social majority. Overall, this communication agenda paid attention to the symbolic construction of an imagined community fighting the virus; direct and implicit references to family and religion were pivotal in maintaining its coherence.

This article has analysed the cultural responses to the COVID-19 pandemic in Greece during the first wave, discussing the endorsement of the lockdown and its limits beyond the obvious explanation that people obeyed it due to fear of the virus. Although the impact of the pandemic in Greece was limited in terms of human losses at that stage, Greeks accepted the restrictions, believing they were essential to protect themselves. The low number of cases and the communication management of the pandemic gave the impression that Greece reacted well. International media applauded the ways in which Greeks faced the pandemic, overturning the dominant discourse of the previous decade during which Greece was discussed as an ineffective country. The government’s communication agenda highlighted this success story cultivating emotions of pride. This resulted in a wave of sympathy for the government, which enjoyed very high levels of popularity. The government achieved consensus for the measures through an emotional vocabulary that utilised the fear about the virus and invested in the appeal of traditional values such as the designation of family as a locus of security. The management of the pandemic inspired solidarity and pride in the public and side-lined the class dimension of the quarantine, designating COVID-19 as a great equaliser and the pandemic as a ‘people versus the virus’ issue. The fact that economic questions remained little discussed, even by the opposition, was evidence of the effectiveness of the government’s communication management at that stage.

The first lockdown ended in late May 2020. Activities gradually restarted. Greece reopened its borders (mostly to EU citizens), illustrating itself as a safe destination and maintaining moderate measures. This initiative aimed to demonstrate that Greece was self-confident in facing the challenge of re-opening. In contrast with Spain, which is also a tourist country, masks were optional in public. Tourists could enter Greece without a recent PCR or antigen test. Avgeropoulos (2021) reveals that this re-opening dissatisfied some epidemiologists (who wished for stricter border controls) and spread the virus throughout the country, which had remained protected in the first wave: 2,931,614 travellers visited Greece in July and August 2020 but only 360,216 (12.8%) were tested. Greece paid a high price for its economic reliance on tourism. From another vantage point, this demonstrated that the narrative of efficiency and pride in conditions of isolation could not succeed forever in a tourism-dependent country such as Greece. Inaugurated in an optimistic speech by Mitsotakis in the famous sunset at Santorini Island under the slogan ‘Greek summer is a state of mind’, this opening enjoyed piecemeal success: Greece earned 4.28 billion Euros from tourism in 2020, experiencing a loss of 76.5 percent in comparison with 2019 (businessdaily.gr, 2021).

Greeks changed their lifestyles in the summer of 2020: most avoided socialising in bars or restaurants and travelling; 54.5 percent argued that their psychological condition was worse than in 2019 (Georgakopoulos, 2021: 42). After September the situation worsened; the government decided on a new lockdown in early November 2020. During the second lockdown, the number of victims was higher: approximately 1,500 deaths in October 2020 and 7,000 deaths in March 2021. The political consensus was limited as opposition parties criticised the government for not radically strengthening the national health system during the summer. As in the United Kingdom, where support for the government declined as the pandemic developed (Newton, 2020: 508), Greek public opinion became increasingly dissatisfied: only 43 percent declared themselves satisfied with how the government handled the pandemic in November 2020 compared with 84 percent the previous May (*Ta Nea*, 2020c). Nevertheless, largely due to the successful management of the first wave, the government still enjoyed popularity in surveys. The success story of the first wave highlighted Greece as a positive international story in the battle against COVID-19. This did not last for a long time, but it left its mark on the Greek political and cultural landscape of the pandemic.

## Notes

1. For Italy, see Alicandro et al. (2020).

2. Beyond Greece, relations between individualism and the endorsement of hygienic measures imposed by the state seem fluid. In the COVID-19 crisis, societies classified as individualistic, such as the United States, Australia, the United Kingdom, Canada, the Netherlands, New Zealand, Ireland, Germany or South Africa (Rothwell, 2016: 59) responded in different ways. Indicatively, in New Zealand, measures received mild reactions, whereas in Germany and the United States, a large number of people saw restrictions as a threat to individual freedom.

3. Furthermore, 17 percent disagreed with this opinion and 27.3 percent believed that the result would have been more or less the same (*Ta Nea*, 2020b).

4. According to Pulse’s monthly surveys, 38 percent preferred New Democracy in November and December 2019 and 45 and 42.5 percent preferred this party in April and May 2020, respectively; 24, 25, 22 and 22 percent, respectively, preferred SYRIZA.

5. For example, see his interview to Antenna TV (2019).

6. Available on YouTube (2020).
